# Notch regulates Th17 differentiation and controls trafficking of IL-17 and metabolic regulators within Th17 cells in a context-dependent manner

**DOI:** 10.1038/srep39117

**Published:** 2016-12-15

**Authors:** Manuel Coutaz, Benjamin P. Hurrell, Floriane Auderset, Haiping Wang, Stefanie Siegert, Gerard Eberl, Ping-Chih Ho, Freddy Radtke, Fabienne Tacchini-Cottier

**Affiliations:** 1 Department of Biochemistry, WHO-Immunology Research and Training Center, University of Lausanne, Switzerland; 2 Department of Fundamental Oncology, Ludwig Center for Cancer Research, University of Lausanne, Switzerland; 3 Institut Pasteur, INSERM U1224, Microenvironment and Immunity Unit, Paris, France; 4 Ecole Polytechnique Fédérale de Lausanne, School of Life Sciences, Swiss Institute of Experimental Cancer Research, Lausanne, Switzerland

## Abstract

Th17 cells play critical roles in host defense and autoimmunity. Emerging data support a role for Notch signaling in Th17 cell differentiation but whether it is a positive or negative regulator remains unclear. We report here that T cell-specific deletion of Notch receptors enhances Th17 cell differentiation in the gut, with a corresponding increase in IL-17 secretion. An increase in Th17 cell frequency was similarly observed following immunization of T cell specific Notch mutant mice with OVA/CFA. However, in this setting, Th17 cytokine secretion was impaired, and increased intracellular retention of IL-17 was observed. Intracellular IL-17 co-localized with the CD71 iron transporter in the draining lymph node of both control and Notch-deficient Th17 cells. Immunization induced CD71 surface expression in control, but not in Notch-deficient Th17 cells, revealing defective CD71 intracellular transport in absence of Notch signaling. Moreover, Notch receptor deficient Th17 cells had impaired mTORC2 activity. These data reveal a context-dependent impact of Notch on vesicular transport during high metabolic demand suggesting a role for Notch signaling in the bridging of T cell metabolic demands and effector functions. Collectively, our findings indicate a prominent regulatory role for Notch signaling in the fine-tuning of Th17 cell differentiation and effector function.

Notch signaling is an evolutionarily conserved cell-to-cell signaling cascade involved in many cell fate decision processes, including early T cell development in the thymus and modulation of peripheral T cell differentiation^1^,^2^. Mammals contain four Notch receptors (Notch1-4) that are activated by engagement of five transmembrane-bound ligands (Delta-like (Dll) 1, 3, 4 and Jagged 1, 2). Interaction of Notch receptors with their ligands leads to the release by proteolytic cleavage of the active intracellular domain of Notch (NICD). NICD translocates into the nucleus, where it forms a complex with recombination signal-binding protein-J (RBP-Jκ). The NICD/RBP-Jκ complex recruits co-activators that facilitate the transcriptional activation of Notch target genes. Alternatively, Notch can also mediate RBP-J independent signaling by interacting with NF-κB^3^,^4^ or TGF-β family members^5^,^6^ which is referred to as non-canonical signaling. Among the factors influencing Th cell differentiation, Notch signaling has been reported to play a role in the differentiation and function of multiple Th cell subsets, such as Th1, Th2, T_regs_ (reviewed in refs 1,7 and 8), and in the more recently described Th9 and Tfh cells^5^,^9^.

Naïve CD4^+^ T cells differentiate into specialized T helper cell (Th) subsets characterized by their expression of transcription factors, the secretion of selected cytokines and distinct effector functions. Among these, Th17 cells play an essential role in the containment of commensals and pathogenic microorganisms in the gastrointestinal tract. Intestinal symbionts, and in particular segmented filamentous bacteria (SFB) contribute to Th17 cell differentiation in the intestinal *lamina propria* where these cells are abundant. Th17 cells are also involved in the control of extracellular bacteria and fungal infections in other mucosal tissues and they can play pathogenic roles in autoimmune diseases (reviewed in ref. 10).

Th17 cells are defined by the expression of the RORγt transcription factor and their secretion of inflammatory cytokines including IL-17A/IL-17F, IL-22, GM-CSF and depending on the context, IFN-γ^11^. The nuclear hormone receptor RORγt, a key transcription factor driving Th17 cell differentiation^12^,^13^ is also involved in the differentiation of ILC3s, an innate lymphoid cell population that also secretes IL-17 and IL-22 (reviewed in ref. 14).

In addition to Th17 cells, FOXP3^+^ regulatory T cells are also present in the intestine and the presence of TGF-β decides between one or the other Th subset^15^,^16^,^17^. Recently, RORγt was also shown to be expressed in a subset of FOXP3^+^ tissue regulatory T cells residing mostly in the colon and to a lesser extent in the small intestine. Differentiation of these RORγt^+^ FOXP3^+^ regulatory T cells is induced by symbionts^18^,^19^. These cells do not express Helios, a marker of thymus-derived T_reg_ cells^20^ and thus differ from the intestinal RORγt^−^ T_reg_ which express Helios and the GATA3 transcription factor^21^,^22^. RORγt^+^ T_reg_ cells do not secrete IL-17 but secrete IL-10. The pathways inducing RORγt^+^ T_reg_ cells appear similar to those leading to the differentiation of Th17 cells^18^,^19^.

The differentiation of Th17 cells is complex, requires fine regulation, and is thought to be balanced with that of T_reg_ cells. Notch signaling can modulate the differentiation of several Th cell subsets^8^,^23^,^24^. However how Notch modulates Th cell subset differentiation mechanistically needs further investigation.

The impact of Notch signaling on complex T cell interactions taking place during the differentiation of Th17 cells and RORγt^+^ T_reg_ cells in gut homeostasis has not been previously investigated.

In this study, we selectively ablated Notch receptors on peripheral T cells to explore the regulatory role of the Notch pathway on the differentiation and effector function of Th17 cells and RORγt^+^ T_reg_ cells in the gut. Furthermore, we compared the impact of Notch receptor ablation on gut T cells with that occurring following the higher metabolic demand that takes place in draining lymph node T cells following immunization.

## Results

### Notch receptor expression on Th17 cells

**Figure 1:**
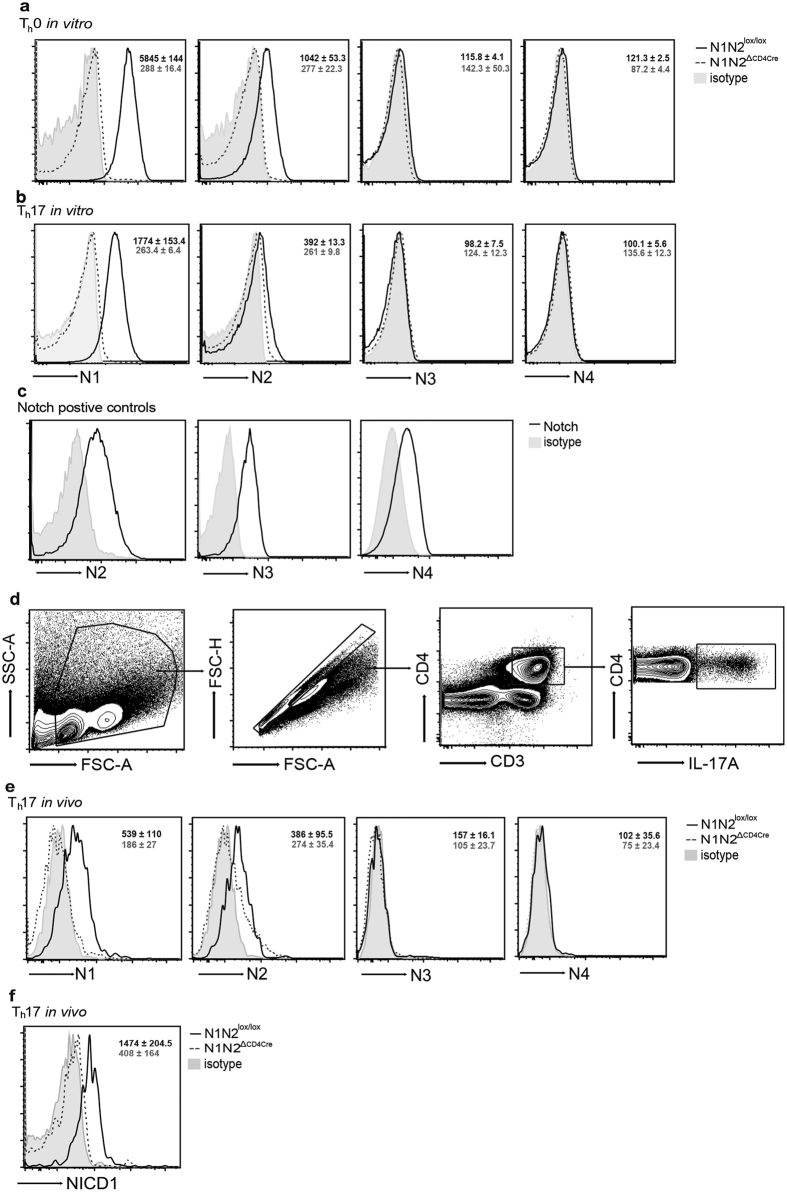
Notch1 and Notch2 are selectively expressed on Th17 cells. CD4^+^ CD62L^+^ T cells isolated from naive control (N1N2^lox/lox^) and N1N2^ΔCD4Cre^ mice were cultured on plate bound α-CD3/α-CD28 (1.0 μg/ml) in absence (Th0 *in vitro*), or in presence, of Th17-cell polarizing conditions (Th17 *in vitro*) during 72 hours. Cells were stained with N1-N4 receptor surface mAbs and analyzed by flow cytometry. Representative histograms of N1-N4 receptor surface expression in live-gated DAPI^−^ CD4^+^ T cells grown following (**a**) Th0 and (**b**) Th17 cell differentiation (Th17) are shown. (**c**) As positive controls, B220^+^ CD4^−^ B cells were stained with N2, CD4^−^CD8^−^ CD25^+^ thymic cells were stained with N3 and CD11c^+^ CD8^+^ DC were stained with N4 mAbs. (**d**) Gating flow cytometry strategy for Th17 cells *ex vivo*. (**e**) Representative histograms of N1-N4 receptor surface expression in gated-IL-17A^+^ CD4^+^ CD3^+^ T cells in naive mLNs from control and N1N2^ΔCD4Cre^ mice. (**f**) Representative histograms of NICD1 expression in gated-IL-17A^+^ CD4^+^ CD3^+^ T cells in mLNs. The MFI ± SEM (n ≥ 3 per group) of Notch receptors or NICD1 expression in both groups of mice is given in the upper right. Results are representative of 3 independent experiments.

To investigate Notch receptor expression during Th17 cell differentiation *in vitro*, isolated naïve CD4^+^ CD62L^+^ T cells from control (N1N2^lox/lox^) and N1N2^ΔCD4Cre^ mice were stained with Notch-specific mAbs. Of the four Notch receptors, only Notch1 (N1) and Notch2 (N2) expression was detected in Th0 cells activated in the presence of anti-CD3/anti-CD28, in line with previous studies^25^. A predominant expression of N1 over N2 was observed following Th17 cell differentiation (Fig. 1a,b). IL-2 increases N1 expression^25^,^26^ and the slightly reduced N1 and N2 expression levels detected in Th17 compared to Th0 cells correlated with reduced levels of IL-2 secretion usually observed during Th17-polarizing conditions^27^. Importantly, no compensatory expression of Notch3 (N3) and Notch4 (N4) was observed on N1N2^ΔCD4Cre^ CD4^+^ T cells in Th0 and Th17 cells (Fig. 1a,b), these antibodies, however, stained positive control cells (Fig. 1c). These data show that N1, and to a lesser extent N2, are the two receptors expressed during Th17 cell differentiation *in vitro.*

To further investigate if Notch receptors were similarly expressed on Th17 cells *in vivo*, we analyzed the expression of Notch receptors on gut associated T cells derived from mesenteric lymph nodes (mLNs), a site where Th17 cells are detectable in naïve mice. Single-cell suspensions from control and N1N2^ΔCD4Cre^ mLNs were stimulated with PMA/Ionomycin, stained with CD4 and IL-17A to gate on Th17 cells (Fig. 1d), and with Notch-specific mAbs (Fig. 1d). As observed *in vitro*, N1 and N2 were expressed at the cell surface of CD4^+^ IL-17^+^ Th17 cells, with no detectable expression of N3 and N4 on Th17 cells. We further observed a corresponding increase of the Notch1 intracellular domain (NICD1) in mLNs of CD4^+^ IL-17A^+^ cells. As expected, NICD1 expression was not observed in Th17 cells lacking Notch receptors (Fig. 1e). These results show that the Notch signaling pathway is activated in mLN Th17 cells *in vivo.*

### Notch receptors regulate IL-17A mRNA transcription and protein levels in mesenteric lymph node Th17 cells

**Figure 2:**
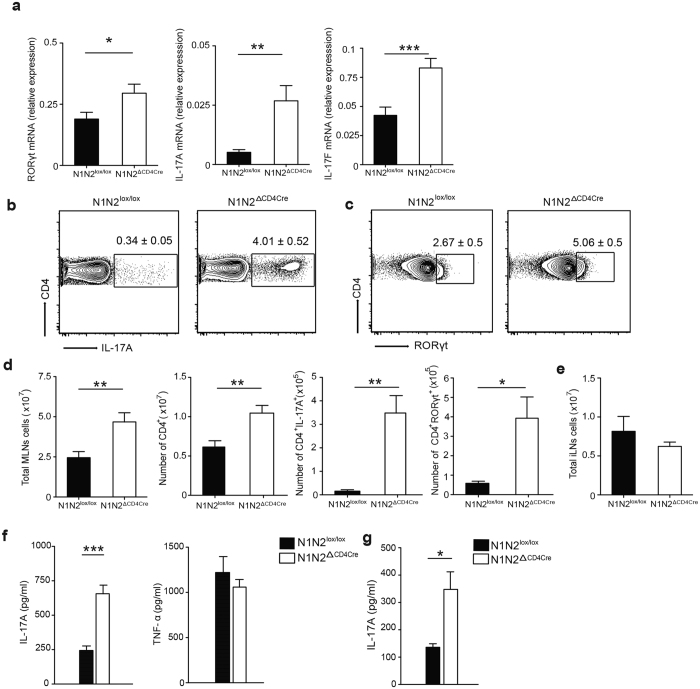
N1 and N2 receptors control IL-17A mRNA transcription and expression in mLNs Th17 cells. (**a**) RORγt, IL-17A, IL-17F mRNA levels were analyzed by quantitative RT-PCR in isolated mLN CD4^+^ T cells of control and N1N2^ΔCD4Cre^ naïve mice. (**b**) Intracellular IL-17A levels were assessed in PMA/Ionomycin restimulated mLNs CD4^+^ T cells. Numbers in representative flow cytometry plots show the mean frequency of IL-17A^+^ within CD4^+^ T cells ± SEM. (**c**) RORγt^+^ expression was analyzed in mLNs CD4^+^ T cells. Flow cytometry plots show the mean frequency of RORγt^+^ within CD4^+^ T cells ± SEM. (**d**) Total number of mLNs, CD4^+^, CD4^+^ IL-17A^+^ and RORγt^+^-CD4^+^ cells are shown ± SEM. (**e**) Total number of cells in naive iLNs cells are shown. (**f**) Total mLN cells or (**g**) isolated mLNs CD4^+^ T cells were restimulated for 72 hours on plate bound α-CD3/α-CD28 (2.5 μg/ml). Mean cytokine levels ± SEM of IL-17A, TNF-α analyzed by ELISA in cell-free supernatants are shown. Mean values ± SEM (n ≥ 3 per group) are shown. Results are representative of 4 independent experiments (n ≥ 3 mice per group); *p < 0.05, **p < 0.01, ***p < 0.001 *versus* control, unpaired, two-tailed student’s test.

To investigate the importance of Notch signaling in Th17 cell differentiation within gut associated mLNs, we analyzed mRNA levels in sorted CD4^+^ T cells isolated from mLNs of naïve N1N2^ΔCD4Cre^ and littermate control mice. An increase in *Rorγt* mRNA levels and a marked increase in *il-17A* and *il-17F* mRNA levels were observed in CD4^+^ T cells that do no express Notch receptors (Fig. 2a). These data are in line with a higher frequency of IL-17A^+^ and RORγt^+^ expressing CD4^+^ Th17 cells detected by flow cytometry analysis in N1N2^ΔCD4Cre^ compared to control mLN cells (Fig. 2b,c). Mice lacking Notch expression in their T cells had an overall larger number of total and CD4^+^ T cells in their mLNs, resulting in an overall higher number of Th17 cells expressing IL-17A and RORγt (Fig. 2d). Of note, cell number of other peripheral lymph nodes such as inguinal lymph node (iLNs) did not differ between N1N2^ΔCD4Cre^ and control mice (Fig. 2e).

To analyze cytokine secretion by mLN Th17 cells, N1N2^ΔCD4Cre^ and control mLN cells were isolated and their TCR activated *ex vivo*. 72 hours later, cytokine levels were analyzed in cell-free supernatant. In agreement with the higher frequency of Th17 cells detected by flow cytometry, higher levels of IL-17A were observed in mLN total cells, or CD4^+^ T cells of Notch-deficient mice, compared to those observed in control mLN cells. In contrast, the levels of TNF-α, a cytokine not produced by Th17 cells, was not affected (Fig. 2f,g). Taken together, these results suggest that Notch receptors are essential in the negative regulation of Th17 cell differentiation in mLNs.

### Notch receptors regulate Th17 and RORγt^+^ T_reg_ cell differentiation in the gut

**Figure 3:**
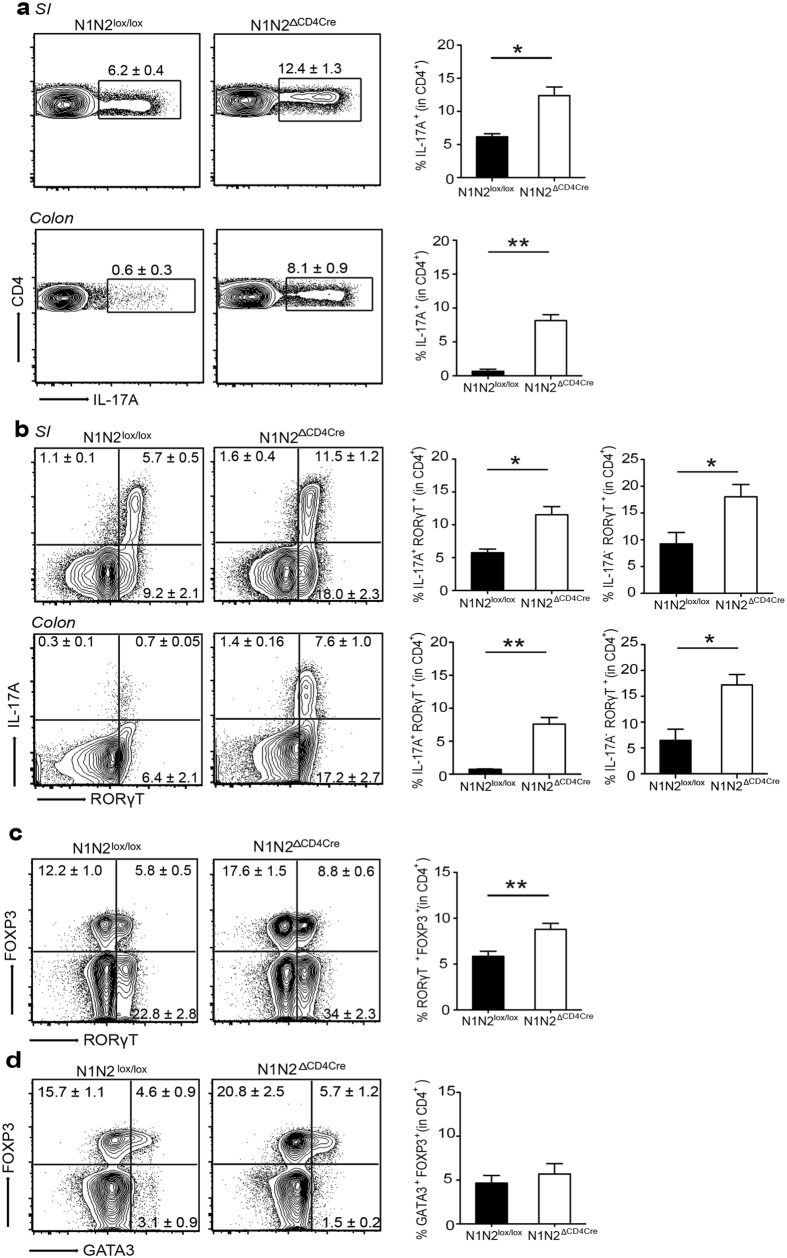
Expression of N1 and N2 receptors regulate Th17 and RORγt ^+^FOXP3^+^; FOXP3^+^ T_reg_ cell frequencies in the gut. Lymphocytes were isolated from the LP of small intestine (SI) and colon of naïve N1N2^lox/lox^ and N1N2^CD4Cre^ mice. IL-17A frequency in CD45^+^ CD4^+^ CD3^+^ cells was assessed by flow cytometry on PMA/Ionomycin stimulated cells in the SI and colon. (**a**) Numbers in representative flow cytometry plots as well as histograms on the right show the mean frequency of IL-17A^+^ in gated CD45^+^ CD4^+^ CD3^+^ T cells in the SI and colon. (**b**) Representative flow cytometry plots and histrogram bars of the frequency of IL-17A^+^ RORγt^+^ and IL-17A^−^ RORγt^+^ cells gated on CD45^+^ CD4^+^ CD3^+^ T cells in the SI and colon (**c**) Representative flow cytometry and histogram of the mean frequency of RORγt ^+^FOXP3^+^ in CD45^+^ CD4^+^ CD3^+^ T_reg_ cells present in the SI is shown. (**d**) Representative flow cytometry plots and histrograms show the mean frequency of FOXP3^+^GATA3^+^ in CD45^+^ CD4^+^ CD3^+^ T_reg_ cells in the SI. Mean values ± SEM (n ≥ 3 per group) are shown. Data are shown as a pool of ≥3 mice per group; *p < 0.05, **p < 0.01 *versus* control, unpaired, two-tailed student’s test.

Th17 cells preferentially accumulate in response to symbionts in the intestine^28^. Accordingly, a population of IL-17 expressing cells was detected in the lamina propria of the small intestine and, to a lesser extent, in that of the colon of control mice. CD4^+^ IL-17^+^ expressing T cell frequency in these organs was strikingly increased in N1N2^ΔCD4Cre^ mice (Fig. 3a). Correspondingly, an increased frequency of IL-17^+^ RORγt^+^ Th17 cells was observed in the small intestine and colon of T cells deficient for Notch signaling compared to the frequency observed in similar organs of control mice (Fig. 3b). The frequency of CD4^+^ RORγt^+^ IL-17^−^ T cells observed was significantly higher than that of CD4^+^ RORγt^+^ IL-17^+^ Th17 cells. RORγt^+^ FOXP3^+^ T_reg_ cells have been recently reported in the colon and small intestine^18^,^19^. We observed an increase of this latter population in N1N2^ΔCD4Cre^ T_reg_ cells of the small intestine lamina propria (Fig. 3c). Another population of T_reg_ cells originating from the thymus, that expresses the GATA3 transcription factor, is also present in the gut. The absence of Notch receptors on peripheral T cells did not have any impact on this population in the small intestine (Fig. 3d). Similar data were obtained in the colon (Fig. S1). Collectively, our results reveal that Notch receptor mediated signaling negatively regulates the differentiation of Th17 and RORγt^+^ FOXP^+^ T_reg_ cells in the gut at homeostasis.

### Context-dependent regulation of Th17 cytokine secretion by Notch

**Figure 4:**
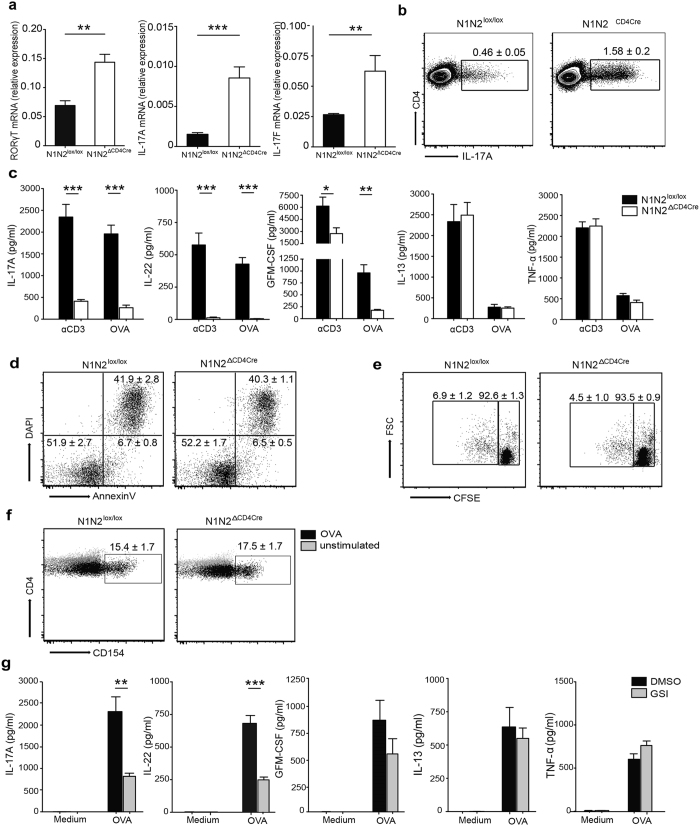
The absence of Notch on T cells affects the secretion of Th17 cell associated cytokines after immunization with OVA emulsified in CFA. Nine days post immunization with OVA/CFA, RORγt, IL-17A and IL-17F, mRNA levels were analyzed by quantitative RT-PCR in isolated CD4^+^ T cells from control and N1N2^ΔCD4Cre^ dLNs. (**a**) Intracellular IL-17A frequency was analyzed following PMA/Ionomycin. FACS plots show the frequency of IL-17A^+^ RORγt^+^ within CD4^+^ T cells ± SEM. Mean values ± SEM (n ≥ 3 per group) are shown (**b**). CD4^+^ T cells isolated from control and N1N2^ΔCD4Cre^ immunized mice were restimulated for 72 hours with plate bound α-CD3 (0.5 μg/ml) or with OVA in presence of irradiated splenocytes. (**c**) Mean cytokine levels ± SEM of IL-17A, IL-22, GM-CSF and IL-13, TNF-α analyzed by ELISA in cell-free supernatants are shown. (**d**) Apoptosis of dLN CD4^+^ T cells was assessed by FACS analysis following AnnexinV and DAPI staining 72 hours after OVA restimulation *in vitro*. Numbers in quadrants represent the mean frequency within CD4^+^ T cells ± SEM. (**e**) Proliferation in dLN live-gated DAPI^−^ CD4^+^ was analyzed by FACS using CFSE staining 96 hours after OVA restimulation *in vitro*. Numbers in FACS plots represent mean percentage of CFSE^+^ dividing cells in CD4^+^ T cells ± SEM. (**f**) Antigen-specific CD4^+^ T cells were detected by flow cytometry analysis following CD154 staining after *in vitro* O/N OVA restimulation and compared to unstimulated CD4^+^ T cells. Numbers in FACS plots represent mean percentage in dLN live-gated DAPI^−^ CD4^+^ CD154^+^  ± SEM. (**g**) dLNs WT CD4^+^ T cells were pre-treated with DMSO or GSI (DAPT) during 30 min and restimulated in the presence of irradiated splenocytes for 72 hours without (Medium) or with OVA. Mean cytokine levels ± SEM of IL-17A, IL-22, GM-CSF and IL-13, TNF-α are shown. Mean values ± SEM (n ≥ 3 per group) are shown. Results are representative of 4 independent experiments (n ≥ 3 mice per group); *p < 0.05, **p < 0.01, ***p < 0.001 *versus* control, unpaired, two-tailed student’s test.

To investigate if Notch signaling is playing a similar role on Th17 differentiation in the context of a stronger stimulus such as following immunization, mice were injected with OVA emulsified in CFA, an adjuvant inducing differentiation of Th17 cells. Nine days after injection, CD4^+^ T cells were isolated from dLNs and processed for analysis. Th17 cells represent only a small percentage of the total CD4^+^ T cells analyzed for mRNA expression, thus even if the relative mRNA levels appear low, a significant increase in RORγt, IL-17A, IL-17F and mRNA levels was observed in CD4^+^ T cells lacking Notch receptor expression (Fig. 4a). Correspondingly, a higher frequency and number of intracellular IL-17A^+^ CD4^+^ T cells, and RORγt^+^ CD4^+^ T cells, were observed by flow cytometry analysis of dLN cells following PMA-ionomycin (Fig. 4b and Fig. S2 b,c), or TCR stimulation (Fig. S2a). These data indicate that, as observed in the gut, N1 and N2 receptors control T_h_17 cell differentiation following immunization.

To further analyze Th17 effector function the levels of Th17-associated cytokines secreted following OVA/CFA immunization, dLN CD4^+^ T cells were isolated from control and N1N2^ΔCD4Cre^ mice 9 days after immunization and restimulated, or not, *ex vivo,* with αCD3 or OVA in the presence of irradiated splenic cells. Th17-secreted cytokines were then assessed in cell-free supernatant. Surprisingly, and in contrast to the elevated levels of intracellular IL-17 observed in N1N2^ΔCD4Cre^ CD4^+^ T dLN cells, a marked decrease in the levels of Th17 cytokines IL-17A, IL-22 and GM-CSF were observed in the supernatant of TCR, or antigen-specific-restimulated N1N2^ΔCD4Cre^ CD4^+^ T cells, compared to that observed in control dLN T cells. Of note, secretion of IL-13 and TNF-α, two cytokines not secreted by Th17 cells, did not vary between Notch-deficient and control CD4^+^ T cells (Fig. 4c). Following immunization with OVA/CFA, the majority of Th cells induced in N1N2lox/lox mice are CD4^+^ IFNγ^+^ Th17 cells. IL-17^+^ IFNγ^+^ double producer T cells are not detectable, rather a small proportion of CD4^+^ IFNγ^+^ Th1 cells are induced. Among these, increased IFNγ expression is observed in N1N2^ΔCD4Cre^ T cells as assessed by flow cytometry, despite markedly decreased IFNγ secretion by these Th1 cells upon OVA restimulation (Fig. S3). These results are in line with a previous study also showing increased retained IFNγ intracellular levels but defective IFNγ secretion by Notch-deficient Th1 cells^29^.

To gain further insight into the mechanism by which Notch receptors control Th17 cytokine secretion, we first investigated if the observed decrease in Th17 cytokine secretion could result from increased apoptosis or reduced CD4^+^ T cell proliferation upon OVA restimulation *ex vivo*. Similar levels of live (Annexin V^−^/DAPI^−^) and apoptotic/necrotic (Annexin V^+^/DAPI^+^) cells (Fig. 4d), and comparable CFSE staining were observed between N1N2^ΔCD4Cre^ and control T cells as analyzed by flow cytometry (Fig. 4e), denoting similar proliferation and apoptosis status in Notch deficient and control group of cells. Moreover, the difference in cytokine secretion does not result from the failure of N1N2^ΔCD4Cre^ CD4^+^ T cells to respond to OVA, as these cells expressed similar levels of CD154, an indication that comparable levels of antigen-specific CD4^+^ T cell activation^30^ occurs in N1N2^ΔCD4Cre^ and N1N2^lox/lox^ CD4^+^ T cells. To investigate if the observed decrease in cytokine release was dependent on Notch receptor cleavage, dLN CD4^+^ T cells from immunized N1N2^lox/lox^ mice were treated with γ-secretase inhibitor (GSI) and then stimulated *ex vivo* with OVA. A selective decrease in IL-17A, IL-22 and GM-CSF cytokine secretion was observed with no impact on IL-13 and TNF-α secretion (Fig. 4g). These data suggest that proteolytic activation of the Notch receptors is necessary for modulating Th17 cytokine secretion.

Furthermore, kinetics of cell viability was analyzed following *ex vivo* stimulation with OVA. The latter revealed no differences between control and N1N2 ^ΔCD4Cre^ CD4^+^ T cells (data not shown). Taken together, our results show that Notch regulates Th17 differentiation and that depending on the context, Notch receptor signaling regulates secretion of IL-17A and other Th17-related cytokines.

### Notch receptor deficiency on Th17 cells impacts the cytokine secretory pathway

**Figure 5:**
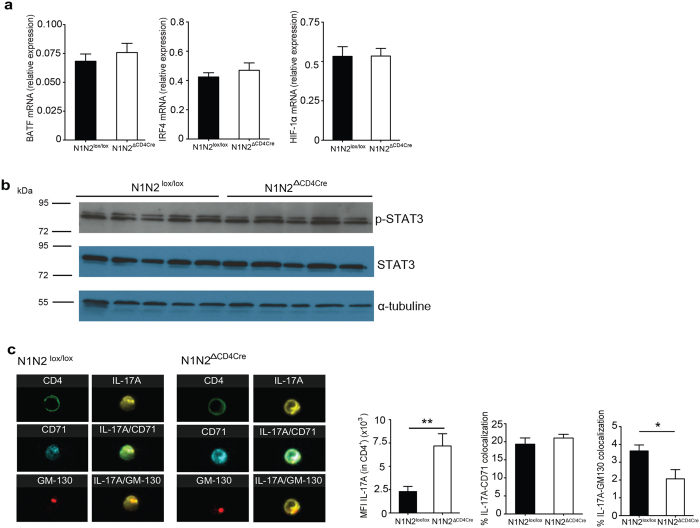
Impact of Notch-deficiency on regulation of IL-17 transcription factors and IL-17 intracellular localization. Nine days post immunization (p.i.) with OVA/CFA, BATF, IRF4, HIF-1α mRNA expression was analyzed by quantitative RT-PCR on isolated CD4^+^ T cells from control and N1N2^ΔCD4Cre^ dLNs. (**a**) CD4^+^ T cells from control and N1N2^ΔCD4Cre^ dLNs were isolated, lysed and western bolt performed using anti p-STAT3 (Tyr705), STAT3, and α-tubulin mAbs. (**b**) Sorted control and N1N2^ΔCD4Cre^ CD4^+^ T cells were restimulated for 7 hours with PMA/Ionomycin to assess IL-17A subcellular distribution in Th17 cells. Representative pictures of colocalization between IL-17A, CD71 (early and recycling endosomes) and between IL-17A and GM130 (Golgi) staining as analyzed by the ImageStream are shown (60x). MFI of IL-17A within CD4^+^, the percentage of colocalization with the indicated antibodies (CD71, GM130) within IL-17A^+^ CD4^+^ are shown. Mean values ± SEM (n ≥ 3 per group) are shown (**e**). Data are representative of 2 independent experiments (n ≥ 3 per group) are shown; *p < 0.05, **p < 0.01 *versus* control, unpaired, two-tailed student’s test.

In the absence of Notch receptor and signaling on T cells, we report here increased expression of RORγt, the key Th17 transcription factor. BATF and IRF4 are transcription factors that are positive regulators of RORγt and IL-17 expression^31^,^32^. To investigate if Notch signaling acted at this level, we analyzed the impact of the absence of Notch on their mRNA levels in dLN CD4^+^ T cells of OVA/CFA immunized mice. A difference in BATF and IRF4 mRNA levels was not observed (Fig. 5a), suggesting that Notch is not acting at this level in Th17 cells. The mammalian target of rapamycin complex (mTOR) signaling has been linked to Notch signaling^33^ and mTORC1 positively modulates IL-17 expression through several pathways including the STAT3 and HIF-1α pathways (ref. 34 and reviewed in ref. 35). We did not observe differences in Hif-1α mRNA or STAT3 phosphorylation levels between N1N2^ΔCD4Cre^ and control CD4^+^ T cells (Fig. 5a,b). These results suggest that defective Notch signaling is not impacting Th17 cell differentiation by acting on the mTORC1 and STAT3 pathways.

The reduced levels of IL-17A secretion observed in OVA immunized N1N2^ΔCD4Cre^ CD4^+^ T cells could be related to an impairment of IL-17A cytokine secretion or trafficking. To further determine if IL-17A was aberrantly located in the Golgi or in the early and recycling endosomes, dLN CD4^+^ T cells isolated from control and N1N2^ΔCD4Cre^ mice immunized with OVA/CFA were restimulated with PMA/Ionomycin *ex vivo*. As negative controls, CD4^+^ T cells from immunized IL-17^−/−^ and control mice treated with isotype controls were used to assess the specificity of IL-17A staining (Fig. S4). CD4^+^ Th17 cells were stained with the GM-130 mAb directed against the Golgi and the CD71 mAb labeling early and recycling endosomes. ImageStream analysis confirmed higher mean fluorescence intensity of IL-17A within Notch deficient Th17 cells. IL-17A was detectable in the Golgi and the endosomes (Fig. 5c). In both N1N2^lox/lox^ and N1N2^ΔCD4Cre^ Th17 cells, IL-17A co-localized with CD71 (IL-17A^+^ CD71^+^). The CD71 and Golgi staining appeared similar in Notch deficient or sufficient cells, however, quantification data analysis showed that colocalization between IL-17A and the Golgi (IL-17A^+^ GM130^+^) was slightly decreased in N1N2^ΔCD4Cre^ Th17 cells (Fig. 5c). These data demonstrate that the IL-17A cytokine is properly synthesized in N1N2^ΔCD4Cre^ Th17 cells. Moreover, the small but significantly decreased levels of IL-17A observed in the Golgi of N1N2^ΔCD4Cre^ Th17 cells suggest either that some IL-17A remains in the ER and/or that the elevated levels of intracellular IL-17A cytokines present in secretory vesicles send a negative feedback to the Golgi.

**Figure 6:**
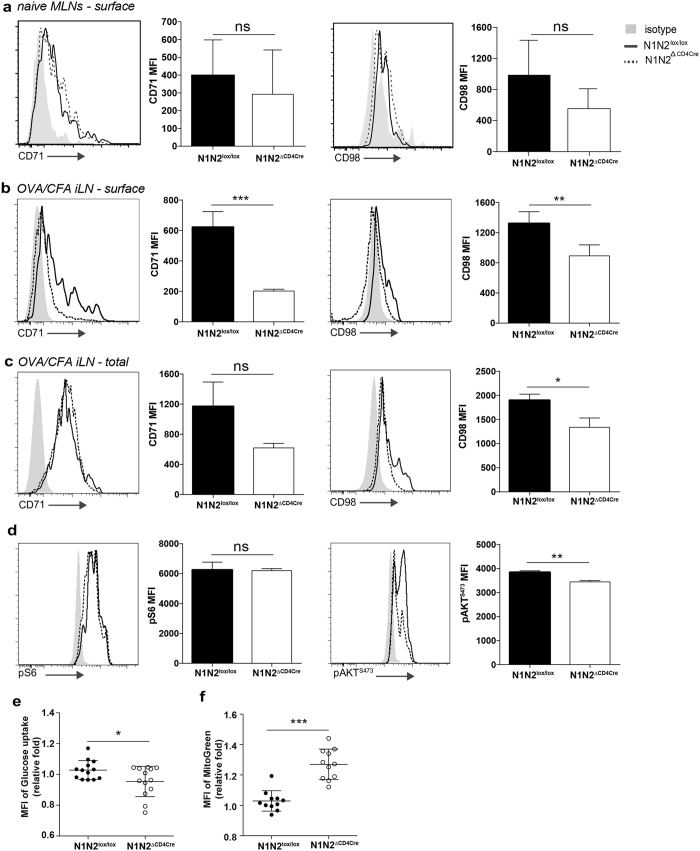
Notch receptor expression regulates metabolic transporter activity within Th17 cells. (**a**) Mesenteric lymph nodes of N1N2^ΔCD4Cre^ and N1N2^lox/lox^ naïve mice were isolated and surface expression of CD71 and CD98 analyzed by flow cytometry, gating on Th17 cells. (**b-f**) Nine days post immunization with OVA/CFA dLN cells were isolated and (**b**) surface expression of CD71 and CD98 analyzed by flow cytometry on Th17 cells (**c**) surface and intracellular levels of CD71 and CD98 (**d**) and p-S6 and p-AKT^S473^ activity were analyzed by flow cytometry gating onTh17 cells. (**e**) 2-NBDG uptake by Th17 cells (**f**) Mitotracker green staining of N1N2^ΔCD4Cre^ and N1N2^lox/lox^ cells, gated on Th17 cells. Results are the combination of 3 independent experiments with 4 mice each per experiment. *p < 0.05, **p < 0.01 *versus* control, unpaired, two-tailed student’s test.

CD71 and CD98 are both nutrient transport proteins whose expression is increased upon T cell activation, reflecting dependence of nutrients for proliferation. Surface expression levels of CD71 and CD98, as analysed by flow cytometry, did not differ between Th17 cells of Notch-deficient, or control mesenteric LNs at homeostasis (Fig. 6a). In contrast, following OVA/CFA immunization, cell surface expression of both transporters was markedly reduced in Notch-deficient Th17 cells, as detected by flow cytometry. These data contrasted with the similar intracellular CD71 levels observed by ImageStream analysis within N1N2^lox/lox^ and N1N2^ΔCD4Cre^ Th17 cells. Following cell permeabilization, which reveals both protein surface and intracellular levels (total), flow cytometry analysis confirmed that similar elevated intracellular levels of CD71 are found in N1N2^lox/lox^ and N1N2^ΔCD4Cre^ Th17 cells. In addition, intracellular levels of CD98 were also increased compared to surface staining in N1N2^ΔCD4Cre^ Th17 cells, but slightly lower levels were detected in N1N2^ΔCD4Cre^ compared to N1N2^lox/lox^ Th17 cells (Fig. 6b).

The retention of CD71 and CD98 in intracellular compartments suggested that other nutrient transporters may not be efficiently transported to the cell membrane. We used the fluorescent glucose analog 2-NBDG to assess cellular glucose uptake in dLN Th17 cells of N1N2^ΔCD4Cre^ or N1N2^lox/lox^ mice immunized 9 days prior with OVA/CFA. We observed a partial but significant decrease in glucose uptake in Th17 cells deprived of Notch expression (Fig. 6e). These data suggest that glucose transporters may not be efficiently transported to the membrane.

The presence of elevated amounts of cytokines and strong TCR stimulation used during *in vitro* Th1 and Th2 differentiation was previously reported to bypass the role of Notch signaling in these artificial conditions^24^,^36^,^37^,^38^,^39^ unlike to what is observed *in vivo* (reviewed in ref. 1). In the same line, a role of Notch during *in vitro* Th17 differentiation was found to be dispensable (Fig. S5). Thus, to investigate a role for Notch signaling in Th17 effector function and its link with metabolism, we further analyzed *in vivo* differentiated Th17 cells after OVA/CFA immunization.

The expression of CD71 and CD98 are controlled by mTOR and their activities further regulate mTOR activity through a positive feedback regulation. Our data on HIF-1mRNA and STAT3 protein expression in immunized CD4^+^ T cells suggested that the mTORC1 pathway is not involved in the regulatory role of Notch on IL-17 release. To further investigate if mTOR components might be involved in Notch regulated IL-17 release, we compared mTORC1 and mTORC2 activity on dLN Th17 cells of mice immunized 9 days before with OVA/CFA. mTORC1 activity was measured by phosphorylation of ribosomal protein 6 (p-S6) and mTORC2 activity by that of Akt phosphorylation at serine 473, two canonical mTOR substrates. In agreement with the data obtained for STAT3 and HIF-1, no difference in p-S6 activity was observed in dLN cells of N1N2^lox/lox^ and N1N2^ΔCD4Cre^ Th17 cells (Fig. 6d left panels) suggesting that Notch is not regulating Th17 differentiation and effector function through mTORC1. In contrast, we observed decreased Akt^S473^ phosphorylation in N1N2^ΔCD4Cre^ compared to N1N2^lox/lox^ Th17 cells (Fig. 6d right panels) which links Notch signaling with mTORC2 activity in immunized Th17 cells.

Downregulation of the mTORc2-Akt pathway in CD8^+^ T cells has been shown to promote mitochondrial biogenesis through a FOXO1-dependent manner^40^,^41^. We therefore analysed if mitochondrial mass, a marker of mitochondrial biogenesis, was impacted by Notch expression on Th17 cells. A significant increase in mitochondrial mass, as assessed by Mitotracker Green labelling, was observed in N1N2^ΔCD4Cre^ Th17 cells as compared to that observed in N1N2^lox/lox^ Th17 cells (Fig. 6f).

Collectively, these data show that Notch signaling controls vesicular trafficking of cytokines and metabolic transporters, as well as Th17 metabolic functions once T cells have strong metabolic needs as observed following immunization.

## Discussion

In this study, we identified a marked impact of Notch receptor expression on the differentiation of Th17 cells in the gut and gut associated lymphoid tissues (GALTs). In naïve mice, Th17 cells preferentially accumulate in the small intestine in response to symbionts including segmented filamentous bacteria (SFB)^42^. Here, we show that the absence of N1 and N2 on T cells resulted in increased frequency of Th17 cells in the mLNs, small intestine and in the colon, compared to that observed in these organs in control mice, suggesting that Notch receptor signaling is a negative modulator of Th17 differentiation in the GALTs at homeostasis. A population of T_reg_ cells expressing RORγt (RORγt^+^ T_reg_) has recently been reported to differentiate in response to microbial symbionts in the colon^18^,^19^. We show here that the Notch signaling pathway is also involved in the differentiation of RORγt^+^ FOXP3^+^ T_reg_ cells, suggesting that Notch regulates similarly Th17 and RORγt-expressing T_reg_ cell differentiation. Whether the type of Notch ligands modulating differentiation of these two cell populations differs remains to be established. Lineage-specific inactivation of Notch receptors, or blocking of Notch signaling in T_reg_ cells by inactivation of RBP-Jκ or Pofut-1 were reported to expand T_reg_ cells and their associated suppressive function in a murine model of graft-versus host diseases^43^,^44^,^45^. We also report here, at homeostasis, an increased frequency of FOXP3^+^ T cells with no impact on the frequency of FOXP3^+^ GATA3^+^ T_reg_ population. These data suggest that Notch distinctly regulates the different T_reg_ populations in the gut of naïve mice.

The involvement of Notch signaling in Th17 cell differentiation has been extensively studied but mostly *in vitro*. There seems to be no consensus on the role of Notch signaling during Th17 cell differentiation. For example, promoter analysis and chromatin immunoprecipitation (ChIP) assays revealed that N1 interacts with both the IL-17 and RORγt promoters *in vitro*, suggesting that these genes could be potential direct Notch targets^46^. Expression of Notch ligands such as DLL4 and Jagged1 on antigen presenting cells (APCs) was shown to enhance Th17 cell differentiation^47^,^48^. Together these results suggested that Notch signaling is a positive modulator of Th17 cell differentiation. Other reports show that pharmacological blockade of Notch signaling leads to a reduction of Th17 cell differentiation^5^,^23^,^46^,^49^. Lower IL-17A levels released by CD4^+^ T cells was observed upon stimulation in mice treated with GSI in experimental multiple sclerosis (EAE), associated with decreased EAE severity^46^. In EAE, T_h_9 cell development was also impaired in the absence of Notch signaling^5^ and reduced disease severity was observed following anti-Dll4 treatment, correlating with a decrease of IFN-γ- and IL-17-producing T cells in both the spleen and central nervous system (CNS)^50^. The impact of canonical Notch signaling on the development of Th17 cells producing either IL-17 (non pathogenic), or both IL-17 and IFNγ^+^, may also differ as recently suggested by a study showing a positive role for canonic Notch signaling in the regulation of IL-23R expression, a receptor critical in the stability of Th17 pathogenic cells^51^. Of note, here we show a negative role for Notch in Th17 cell differentiation following OVA/CFA immunization. This immunization induces the differentiation of Th17 cells secreting IL-17, but not IL-17 and IFNγ. Our data suggest that the modulatory role of Notch signaling may depend on the microenvironment thus favoring the phenotype of developing Th17 cells.

Notch signaling may specifically regulate Th17 cell function through the integration of signals provided by the cytokine signaling pathway such as those of TGF-β or IL-6. Notch was already shown to crosstalk with TGF-β during T_reg_ and Th9 cell differentiation^5^,^6^. TGF-β is furthermore a cytokine present in the gut, which in combination with IL-6 promotes Th17^52^, or at high doses promotes T_reg_ cell differentiation^53^. Thus far, there is no evidence of an interaction between Notch and IL-6 in CD4^+^ T cells. Here we show that integration of IL-6 signaling allows similar STAT3 phosphorylation (the major transducer of IL-6 pathway) in N1N2^ΔCD4Cre^ and N1N2^lox/lox^ dLN CD4^+^ T cells isolated after OVA in CFA immunization. These results reveal that an impact of Notch signaling on IL-6 and STAT3 signaling can be excluded as being major Th17 regulators following OVA/CFA immunization.

No major defect in the subcellular distribution of IL-17A was observed in N1N2^ΔCD4Cre^ T_h_17 cells. T cells differentially secrete cytokines through two distinct release pathways. One pathway controls specific cytokines (IL-2, IFNγ, IL-10) to the immunological synapse where localized cell–cell communication occurs. It is likely that IL-17 is secreted using this pathway. In contrast, the TNF-α pathway differs as this cytokine is secreted in a multidirectional fashion^54^. We previously reported increased levels of intracellular IFNγ but markedly reduced levels of IFN-γ secretion in N1N2^ΔCD4Cre^ Th1 cells following *Leishmania major* infection, an infection inducing Th1 cell differentiation^29^, suggesting that Notch receptor signaling could regulate cytokine release. In line with these data we also measured increased levels of intracellular IFNγ but decreased levels of IFNγ secretion by N1N2^CD4Cre^ Th1 cells following OVA/CFA immunization. We also observed a reduction in IL-17 secretion in this experimental model of infection (unpublished data). Here, following immunization with OVA/CFA, a strong inducer of Th17 cell differentiation, we also report an impact of Notch on Th17 cytokine release. These data suggest that Notch signaling may be involved in the modulation of cytokine transport in Th1 and Th17 cells. The reduced levels of IL-17A and Th17-related cytokine secretion did not appear to result from a general defect in T cell cytokine release. This is due to the observation that other non- Th17 cytokines such TNF and IL-13 were similarly released in control and Notch-deficient T cells. Our results suggest that in order to analyze the mechanisms leading to cytokine production by Th cells, it would be important to analyze both intracellular cytokine levels as well as cytokine production.

Nutrient receptor expression was previously shown to be regulated by Notch in thymocytes^55^. Nutrient uptake is contributing to Th17 differentiation in a process mediated by the activation of the target of rapamycin (TOR), mTORC1, an important regulator of immune functions^56^. However, we did not observe any impact of the absence of Notch on T cell STAT3 phosphorylation, nor on p-S6 activity, both of which are controlled by mTORC1, demonstrating that following OVA/CFA immunization, Notch signaling is not controlling Th17 differentiation *via* the mTORC1 pathway. Notch signaling has been reported to activate mTORC2 and the downstream kinase AKT in a Notch non-canonical pathway during thymocyte differentiation^57^,^58^. It was shown that mTORC2-Akt-FOXO1/3a signaling negatively regulates the differentiation of T_reg_ cells^59^. Furthermore, Notch triggering was shown to increase metabolism and T cell secretion, allowing response to low doses of antigen^60^. We show that the cycling of CD71, the transferrin receptor and that of CD98, a subunit of the L-amino acid transporter are regulated by Notch in Th17 cells following immunization but not at homeostasis. The co-localization of CD71 and IL-17A in Th17 cells implies that IL-17A secretion may be coupled with nutrient uptake. Notch signaling was recently shown to impair epigenetic stability of FOXP3 via the Rictor-AKT-FOXO1 axis^61^. The role of mTORC2 in Th17 differentiation remains unclear with it having either positive^62^,^63^ or negative^64^,^65^ regulatory contributions. Here, we show that the Notch signaling effect on vesicular transport is linked to the mTORC2 pathway. Our data suggest that the impact of Notch on mTORC2 activity in Th17 cells may be context dependent, with differences between homeostasis where metabolic demand is low, and immunization, where metabolic demand is high. Interestingly, down regulation of the mTORC2-Akt pathway in CD8^+^ T cells has been shown to promote mitochondrial biogenesis in a FOXO1-dependent manner^40^,^41^. In line with these results, we also found that following OVA/CFA immunization, Notch-deficient Th17 cells have reduced mTORC2 activity and display a significantly higher mitochondrial mass than Th17 cells from N1N2^lox/lox^ immunized mice. These results indicate a role for Notch signaling in the regulation of mitochondrial metabolism in Th17 cells. Furthermore, glucose uptake ability was only mildly reduced in Notch-deficient Th17 cells, suggesting that Notch signaling may also have a small impact on glucose transporters. Further explorations of Notch signaling on the metabolism and effector function of Th17 cells are warranted.

Our results also suggest that when T cells have strong metabolic needs, Notch signaling, through its control of vesicular trafficking of cytokines and metabolic transporters, might bridge metabolic demands with T cell effector functions.

Taken together, we demonstrate here that Notch negatively modulates Th17 cell differentiation. However, during high metabolic demand this can be counterbalanced by the impact of Notch on intracellular cytokine trafficking, which allows rapid Th17 cytokine release. Strategies aimed at blocking Notch receptors to modulate T_reg_ and/or Th17 effector function may thus have to be thoughtfully considered, taking into consideration the site and magnitude of the immune response.

## Materials and Methods

### Mice

N1N2 mice (referred as N1N2^∆CD4Cre^) were previously described^29^. N1N2^lox/lox^ littermates were used as controls. All mice were on a C57BL/6 genetic background. T cell-specific deletion of Notch was verified by PCR. Mice were bred and maintained under pathogen-free conditions in the animal facility at the Center of Immunity and Infection Lausanne (Epalinges, Switzerland). All animal experimental protocols were approved by the Veterinary office regulations of the State of Vaud, Switzerland and all methods were performed in accordance with the Swiss guidelines and regulations (authorization 1266.4-6 to FTC).

### Cell preparation

Small intestinal and colonic lamina propria mononuclear cells were isolated as previously described^66^. Briefly, Peyer’s Patches were removed, and whole small intestine and colon were opened longitudinally, cut into pieces and incubated for 30 minutes in cold calcium- and magnesium-free PBS containing 30 mM EDTA, washed extensively, and incubated for several rounds in iDMEM (Life Technologies) containing collagenase D (0.7 mg/ml; Roche) and Liberase TL (2.5 mg/ml; Roche) at 37 °C. The remaining intestinal fragments were collected and pressed through 100μm filters (BD Biosciences), mixed with the collected supernatants. Mononuclear cells were separated by a 40/80% (wt/vol) Percoll (Sigma-Aldrich) density gradient and washed prior to staining for flow cytometry.

For cytokine quantification, either single-cell suspensions or isolated CD4^+^ T cells from mLNs by a negative magnetic separation (MACS) using CD4^+^ T cell isolation kit (Miltenyi Biotech) were cultured on plate bound α-CD3ε (clone: 145-2C11) and α-CD28 (clone: 37.51) (eBioscience) (both at 2.5 μg/ml) for 72 hours.

### Immunization

Mice were immunized subcutaneously (s.c.) in the flank with 100 μg per site of OVA protein (Sigma-Aldrich) emulsified in CFA (Sigma-Aldrich, MP Biomedicals) as previously described^67^. 9 days later, inguinal dLNs were harvested. CD4^+^ T cells were isolated from inguinal dLNs using CD4^+^ negative magnetic separation (Miltenyi Biotech) and plated in presence of irradiated syngeneic splenocytes (1500 rad). CD4^+^ T cells were *ex vivo* restimulated without (medium) or with OVA protein (200 μg/well), αCD3ε (0.5 μg/ml), and in some experiments also with (200 μg/well) NP-CGG (Biosearch Technologies) as a control. After 72 hours, supernatants were collected for cytokine analysis. As a control, CFA was injected alone and 9 days later dLN cells were isolated and re-stimulated *ex vivo* with OVA. No IL-17 was detectable in the cell-free supernatant. For drug treatment, isolated CD4^+^ T cells from control immunized mice, were treated during 30 minutes at 37 °C with GSI (10 μM) (DAPT, γ-secretase inhibitor IX) (Merck Milipore) or with DMSO (0.1%) as previously described^46^, and then cultured without or with OVA (200 μg/well) during 72 hours.

### Cytokine quantifications

IL-17A (homodimer), GM-CSF, IL-22 and TNF-α were analyzed using ELISA Ready-SET-GO^**®**^ (eBioscience) and using DuoSet^**®**^ kit (R&D Systems) for IL-13 according to manufacturer’s instructions.

### RNA isolation, cDNA synthesis and real-time PCR

mRNA was extracted with the RNeasy minikit (Qiagen) according to manufacturer’s protocol. cDNA was prepared as previously described^29^. Semi-quantitative real-time PCR was performed using SYBR Green (LightCycler FastStart DNA Master SYBR Green I, Roche) and a LightCycler system (Roche). mRNA was normalized to the relative hypoxanthine phosphoribosyltransferase (HPRT) endogenous mRNA expression as described previously^29^. Primers for IL-17A (5′-TCCAGAAGGCCCTCAGACTA-3′, 5′-CAGGATCTCTTGCTGGATG-3′), IL-17F (5′-AATGCCCTGGTTTTGGTTGAA-3′, 5′-TGCTACTGTTGATGTTGGGAC-3′). RORγt, IRF4, BATF primers were previously described^68^,^69^.

### Antibodies and flow cytometry

The following mAb conjugated were used for surface staining: anti-mouse CD4-FITC, -AlexaFluor700, -PeCy5 (GK1.5, RM4-5), anti-mouse CD45.2-eFluor450 (104), anti-mouse CD3-PeCy5 (145-2C11), anti-mouse CD8-APC, -FITC (53-67), anti-mouse B220-APC, -PE-Texas Red (RA3-6B2), CD71-PE. All conjugates were purchased from eBioscience, except B220-PE-Texas Red from BD Biosciences and CD98 from Biolegend. For intracellular staining, cells were stimulated with PMA (50ng/ml, Sigma-Aldrich), Ionomycin (500ng/ml, Sigma-Aldrich) and GolgiPlug (1 μg/ml, BD Biosciences) for 4 hours at 37 °C or on plate bound α-CD3ε/α-CD28 (both at 2.5 μg/ml) with GolgiPlug (1 μg/ml) for 6 hours at 37 °C as previously described^44^. For intracellular cellular detection of cytokines and transcription factors, cells were fixed and permeabilized using Foxp3/Transcription factor staining buffer staining kit (eBioscience). The following conjugated mAbs were used: anti-mouse IL-17A-PE, -APC (eBio17B7), anti-human/mouse RORγt-PE (AFKJS-9), anti-human/mouse FOXP3-PeCy5 (FJK-16s), anti-human/mouse GATA3-PE (TWAJ) from eBioscience. The isotype controls used (all from eBioscience) were anti-rat IgG1-PE (eBRG1), anti-rat IgG2a-PE (eBR2a), anti-rat IgG1κ-APC (eBRG1) and anti-rat IgG1κ-PeCy5 (eBR2a). For *in vitro* Notch receptor expression, CD4^+^ CD62L^+^ T cells were isolated from naïve spleens and LNs by a negative magnetic separation (MACS) using CD4^+^ T cell isolation kit, followed by a positive selection for CD62L^+^ according to manufacturer’s instructions (both kits from Miltenyi Biotech). CD4^+^ CD62L^+^ T cells were cultured on plate bound α-CD3 and α-CD28 in presence (Th17) or absence (Th0) of Th17-cell polarizing condition as previously described^46^ during 72 hours. Briefly, CD4^+^ CD62L^+^ T cells were isolated from naïve spleens and LNs by a negative magnetic separation (MACS) using CD4^+^ T cell isolation kit, followed by a positive selection for CD62L^+^ according to manufacturer’s instructions (both kits from Miltenyi Biotech). The purity of isolated CD4^+^ CD62L^+^ T cells was up to 90%. Purified cells were cultured on plate-bound α-CD3ε (clone: 145-2C11) and α-CD28 (clone: 37.51) (eBioscience) (1 μg/ml) at a concentration of 1 × 10^6^ cells/ml in IMDM (Life Technologies) in absence (T_h_0) or presence of T_h_17-cell polarizing conditions (T_h_17). T_h_17-cell polarizing conditions were: rIL-6 (20ng/ml) (eBioscience), recombinant human TGF-β1 (3ng/ml) (R&D), anti-IFN-γ (10 μg/ml) (clone: XMG1.2), anti-IL-4 (10 μg/ml) (clone: 11B11).

CD4^+^ T cells were stained with CD4-FITC and with anti-N1, anti-N2 biotinylated mAbs^25^, followed by Streptavidin-PE-Texas Red (Invitrogen, Thermo Fisher Scientific), or with PE-conjugated anti-mouse N3 (HMN3-133) and anti-mouse N4 (HMN4-14) (eBioscience). Dead cells were excluded using DAPI staining (20 μg/ml, Sigma-Aldrich). In the experiments looking at mTOR activity, intracelllular phosphorylated (p) p-Akt^473^, p-S6^235–236^ were detected following 1 h incubation with aCD3/aCD28 (Thermofisher) according to manufactuer’s conditions and cells were fixed and permeabilized using Foxp3/Transcription factor staining buffer staining kit (eBioscience). To analyze cellular glucose uptake rate, 9 days after OVA/CFA immunization, MACS purified CD4^+^ T cells were incubated with 2-NBDG in glucose-free medium at 37 °C (CO2) for 5 minutes. To assess mitochondrial mass Mitotracker Green labeling was used (all mAbs from Invitrogen). CD4^+^ T cells were incubated in RPMI at 37 °C for 15 minutes, washed, stained with surface CD4, CD44 and intracellular IL-17 mAbs and gated CD4^+^ CD44^+^ IL-17^+^ Th17 cells and analyzed by flow cytometry.

For *in vivo* Notch receptor, or NICD1 expression on Th17 cells, single-cell suspensions were stimulated 4 hours with PMA (50ng/ml), Ionomycin (500ng/ml) and GolgiPlug (1 μg/ml), then stained with CD45.2-eFluor450, CD3-PeCy5, CD4-FITC, IL-17A-APC, anti-N1, anti-N2, anti-N3, anti-N4 or anti-human/mouse NICD1-PE (mN1A) (eBioscience) antibodies.

For T cell proliferation assays during *ex vivo* restimulation, dLNs cells were labeled with CFSE (Molecular Probes) and cultured, with or without, OVA protein (200 μg/well). Cells were harvested for flow cytometry after 96 hours. For T cell viability during *ex vivo* restimulation, dLNs cells were cultured during 72 hours with or without OVA. CD4^+^ T cells were stained with DAPI (20 μg/ml) and AnnexinV-FITC (Biolegend). For antigen-specific CD4^+^ T cells assays during *ex vivo* restimulation, dLNs cells were labeled with PE mouse anti-human CD154 (TRAP1) (BD Biosciences) and cultured with OVA and Monensin (0.66 μl/ml, BD Biosciences) during O/N restimulation as previously described^30^. Dead cells were excluded using DAPI (20 μg/ml) staining. All analyses were performed on FACS LSR Fortessa and LSR II flow cytometer (BD Biosciences) and data were processed with FlowJo (Tree Star). The CD98 mAb was from Biolegend, and the CD71 from eBioscience. Intracelllular phosphorylated (p) p-Akt^473^, p-S6^235–236^ were detected following methanol fixation and permeabilization with paraformaldehyde (Cell Signaling mAbs).

### Image Stream flow cytometry

Nine days post injection of OVA in CFA, CD4^+^ T cells from N1N2^lox/lox^ and N1N2^ΔCD4Cre^ inguinal dLNs cells were isolated using CD4^+^ T cell isolation kit (Miltenyi Biotech). 1 × 10^6^ CD4^+^ T cells were restimulated with PMA (50ng/ml), Ionomycin (500ng/ml) and GolgiPlug (1 μg/ml) during 7 hours at 37 °C. CD4^+^ T cells were harvested and surface stained with CD4-FITC (eBioscience), then fixed and permeabilized with fixation/permeabilization kit (BD Biosciences). Permeabilized CD4^+^ T cells were stained with anti-mouse CD71-PeCy5 (Transferrin receptor) (R17217) (eBioscience), IL-17A-PE (eBioscience), anti-mouse GM-130-Alexa647 (35) (BD Biosciences) and DAPI.

Cells were acquired using Inspire software (Amnis) on a 4-laser 12-channel imaging flow cytometer (Image Stream^X^ Mark II) using 40x and 60x magnifications. Prior to each experiment the machine was fully calibrated using ASSIST (Amnis). Per file 20000–100000 single cells were acquired using the 40x objective. In addition a file with 200–300 IL-17A^+^ cells using the 60x magnification was acquired for each sample. Debris and doublets were excluded based on their area and aspect ratio. Single stain controls were acquired as required (all channels on, no brightfield and no side scatter image) and a compensation matrix was calculated and applied to the files using IDEAS (v6.1) software. For analysis, cells in focus (using the “gradient RMS” feature for the brightfield image) and single cells (in a plot using “area” versus “aspect ratio”) were gated. Based on the DAPI area using a 50% threshold mask and the brightfield contrast, apoptotic cells were excluded from the analysis. Using the intensity feature, CD4^+^ cells were gated and within this population the IL-17A^+^ cells. To analyze colocalization with CD71^+^ endosomes and the GM130^+^ Golgi apparatus, respectively, CD4^+^ IL-17A^+^ cells with a bright CD71 or GM130 staining were used. Colocalization was calculated using the “bright detail similarity R3” feature in the cell inside using an erode mask based on the brightfield image, with scores higher than 2.5 considered as colocalizing.

### Western blot

After 9 days post injection of OVA in CFA, CD4^+^ T cells from N1N2^lox/lox^ and N1N2^ΔCD4Cre^ inguinal dLNs cells were isolated using CD4^+^ T cell isolation kit (Miltenyi Biotech). CD4^+^ T cells were lysed in RIPA lysis buffer (Thermo Fischer) complemented with protease/phosphatase inhibitor cocktail (Cell Signaling). Cell lysates were boiled with reducing SDS sample buffer and 25 μg of proteins per sample were analyzed by SDS-PAGE (15%). The nitrocellulose membrane was revealed successively with phospho-STAT3 (Tyr705) (Cell Signaling), STAT3 (Cell Signaling) and α-tubuline (B-5-1-2) (Cell Signaling). To reveal the antibodies, horseradish peroxidase–coupled (HRP), goat anti-rabbit (for pSTAT3) or anti-mouse (for STAT3, α-tubuline) were used (Jackson Immunoresearch). For HRP detection, Amersham ECL (for STAT3, α-tubuline) or Amersham ECL plus (for pSTAT3) prime western blotting reagent were used (GE Healthcare Life Science).

### Statistical analysis

All p-values were determined with GraphPad Prism 5.0c software (GraphPad Software Inc.) using the two-tailed Student’s t-test for unpaired data. The degrees of significance were indicated as: *p < 0.05, **p < 0.01, ***p < 0.001.

## Additional Information

**How to cite this article**: Coutaz, M. *et al*. Notch regulates Th17 differentiation and controls trafficking of IL-17 and metabolic regulators within Th17 cells in a context-dependent manner. *Sci. Rep.* **6**, 39117; doi: 10.1038/srep39117 (2016).

**Publisher's note:** Springer Nature remains neutral with regard to jurisdictional claims in published maps and institutional affiliations.

## Supplementary Material

Supplementary Information
