# Supplementary material for: Notch regulates Th17 differentiation and controls trafficking of IL-17 and metabolic regulators within Th17 cells in a context-dependent manner
Source: Sci Rep. 2016 Dec 15;6:39117. doi: 10.1038/srep39117 (PMC5156918; doi:10.1038/srep39117)
Supplement: Supplementary Information [file srep39117-s1.pdf]

Supplementary information for:

Notch regulates Th17 differentiation and controls trafficking of IL-17 and metabolic regulators within Th17 cells in a context-dependent manner

Manuel Coutaz[1], Benjamin P. Hurrell[1], Floriane Auderset[1], Haiping Wang[2], Stefanie Siegert[2], Gerard Eberl[3], Ping-Chih Ho[2], Freddy Radtke[4] and Fabienne Tacchini-Cottier[1*]

Supplementary Figures:

Figure S1: N1 and N2 receptors control RORγt^{+} T_{reg} cell frequencies in the colon

Lymphocytes were isolated from the *lamina propria* of the colon of naïve N1N2$^{lox/lox}$ and N1N2^{CD4Cre} mice. Representative flow cytometry plots show the mean frequency of RORγt^{+}FOXP3^{+} (a) and Gata3^{+}FOXP3^{+} (b) within CD45^{+} CD4^{+} CD3^{+} T cell from the colon. A representative plot with mean values ± SEM (n≥3 per group) is shown.

Figure S2: Number of CD4^{+}IL-17^{+} and CD4^{+}RORγt^{+} T cells in the colon of N1N2$^{\Delta CD4cre}$ and N1N2$^{lox/lox}$ mice. Nine days post immunization with OVA/CFA, CD4^{+} T cells from N1N2$^{lox/lox}$ and N1N2$^{\Delta CD4Cre}$ dLNs were isolated and stimulated for 7 hours with plate bound α-CD3/α-CD28 (2.5µg/ml). Numbers in representative flow cytometry plots show the mean frequency of IL-17A^{+} within CD4^{+} T cells ± SEM. (a). The total number of dLNs cells, of CD4^{+} T cells and the number of CD4^{+}IL-17A^{+} cells are presented as

histograms ± SEM. (b) The number of $CD4^+ROR\gamma t^+$ cells within dLNs cells is given ± SEM.(c).

Figure S3: Secretion of IFNγ by Th17 cells from dLN cells of OVA/CFA immunized mice.

Nine days post injection with OVA and CFA, dLN $CD4^+$ T cells were isolated from dLN cells of $N1N2^{\Delta CD4Cre}$ or $N1N2^{lox/lox}$ mice and (a) surface stained with anti-$CD4^+$ mAb, and intracelularly stained with IFNγ and IL-17 mAbs and analyzed by flow cytometry. (b) dLN $CD4^+$ T cells were isolated and re-stimulated in the presence of irradiated splenocytes for 72 hours in the presence or absence of OVA, and of plate bound α-CD3 (0.5μg/ml), or NPC-GG as positive and negative controls, respectively. Mean cytokine levels ± SEM of IFNγ analyzed by ELISA in cell-free supernatants are shown.

Figure S4: ImageStream staining controls

Nine days post injection with OVA and CFA, dLN $CD4^+$ T cells were isolated from IL-$17A^{-/-}$ or control dLNs and restimulated for 7 hours with PMA/Ionomycin to assess the specificity of IL-17A staining by ImageStream analysis. Representative pictures of IL-17A GM130 and CD71 staining in IL-$17A^{-/-}$ $CD4^+$ T cells are shown (60x) (a). Representative pictures following staining with RatIgG2a-PE staining, the isotype control for IL-17A mAb staining, CD71 and GM130 staining in control $CD4^+$ T cells as analyzed by the ImageStream are shown. (b) Representative pictures of staining with mAbs against CD4, IL-17A and GM130 staining but in absence of CD71 mAb

staining in control $CD4^+$ T cells are shown. (c) Representative staining obtained following staining with anti CD4, IL-17 and CD71mAbs but in absence of staining with anti-GM130 mAb in control $CD4^+$ T cells are shown (d).

Figure S5: The role of Notch is bypassed under strong *in vitro* Th17 polarization

(a) $CD4^+CD62L^+$ T cells isolated from naive control ($N1N2^{lox/lox}$) and $N1N2^{\Delta CD4Cre}$ mice were cultured on plate bound α-CD3/α-CD28 (1.0µg/ml) in absence (T_h0) or in presence of Th17-cell polarizing conditions (T_h17) during 48 hours. IL-17A, IL-17F and Il-21 and IL-2 cytokine levels were assessed in culture supernatants of Th17 differentiated cells after 24 and 48 hours by ELISA. (b) Representative flow cytometry plot and frequency of $CD4^+IL\text{-}17^+$ cells 48 hours after *in vitro* polarization.

Figure S1

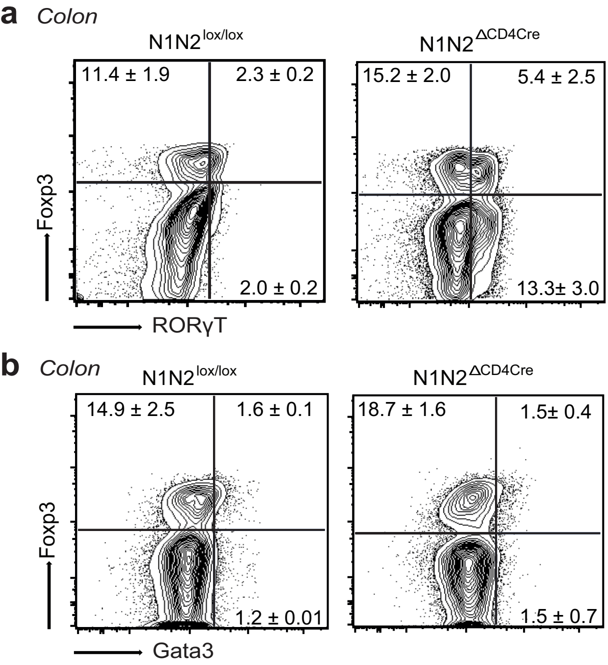
a
Colon
N1N2 lox/lox
N1N2 ΔCD4Cre
11.4 ± 1.9
2.3 ± 0.2
2.0 ± 0.2
15.2 ± 2.0
5.4 ± 2.5
13.3± 3.0
Foxp3
RORγT
b
Colon
N1N2 lox/lox
N1N2 ΔCD4Cre
14.9 ± 2.5
1.6 ± 0.1
1.2 ± 0.01
18.7 ± 1.6
1.5± 0.4
1.5 ± 0.7
Foxp3
Gata3

Figure S2

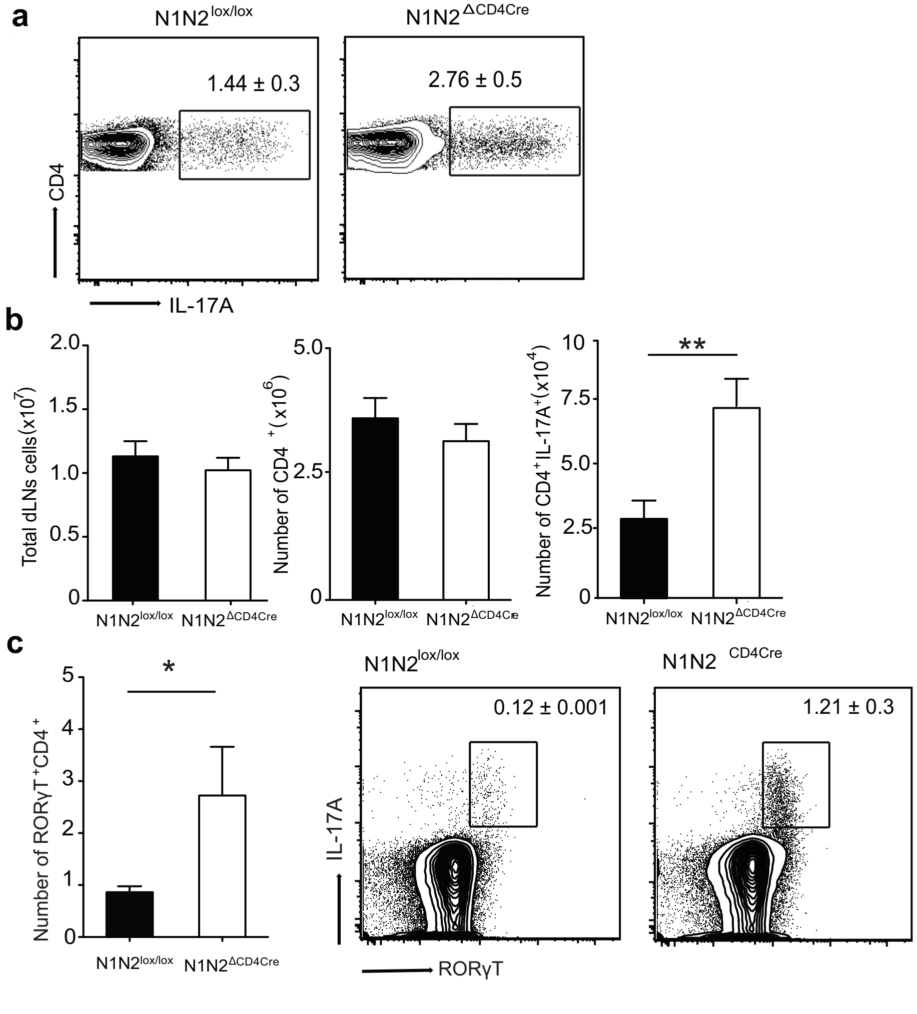
a
N1N2lox/lox
N1N2ΔCD4Cre
1.44 ± 0.3
2.76 ± 0.5
CD4
IL-17A
b
Total dLNs cells(x10^7)
Number of CD4+(x10^6)
Number of CD4+IL-17A+(x10^4)
**
N1N2lox/lox
N1N2ΔCD4Cre
c
*
Number of RORγT+CD4+
N1N2lox/lox
N1N2 CD4Cre
0.12 ± 0.001
1.21 ± 0.3
IL-17A
RORγT

Figure S3

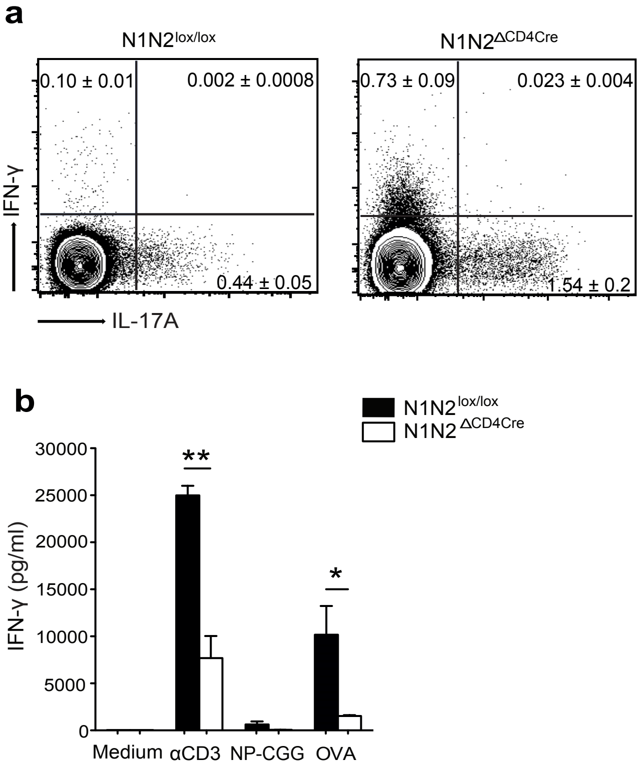
a
N1N2lox/lox
N1N2ΔCD4Cre
0.10 ± 0.01
0.002 ± 0.0008
0.44 ± 0.05
0.73 ± 0.09
0.023 ± 0.004
1.54 ± 0.2
IFN-γ
IL-17A
b
N1N2lox/lox
N1N2ΔCD4Cre
IFN-γ (pg/ml)
30000
25000
20000
15000
10000
5000
0
**
*
Medium
αCD3
NP-CGG
OVA

Figure S4

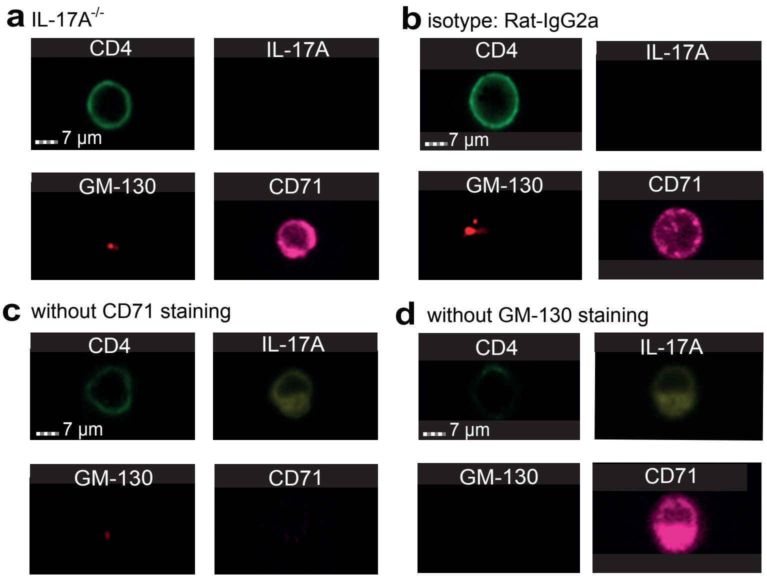

a IL-17A-/-
CD4
IL-17A
7 µm
GM-130
CD71
b isotype: Rat-IgG2a
CD4
IL-17A
7 µm
GM-130
CD71
c without CD71 staining
CD4
IL-17A
7 µm
GM-130
CD71
d without GM-130 staining
CD4
IL-17A
7 µm
GM-130
CD71

Figure S5

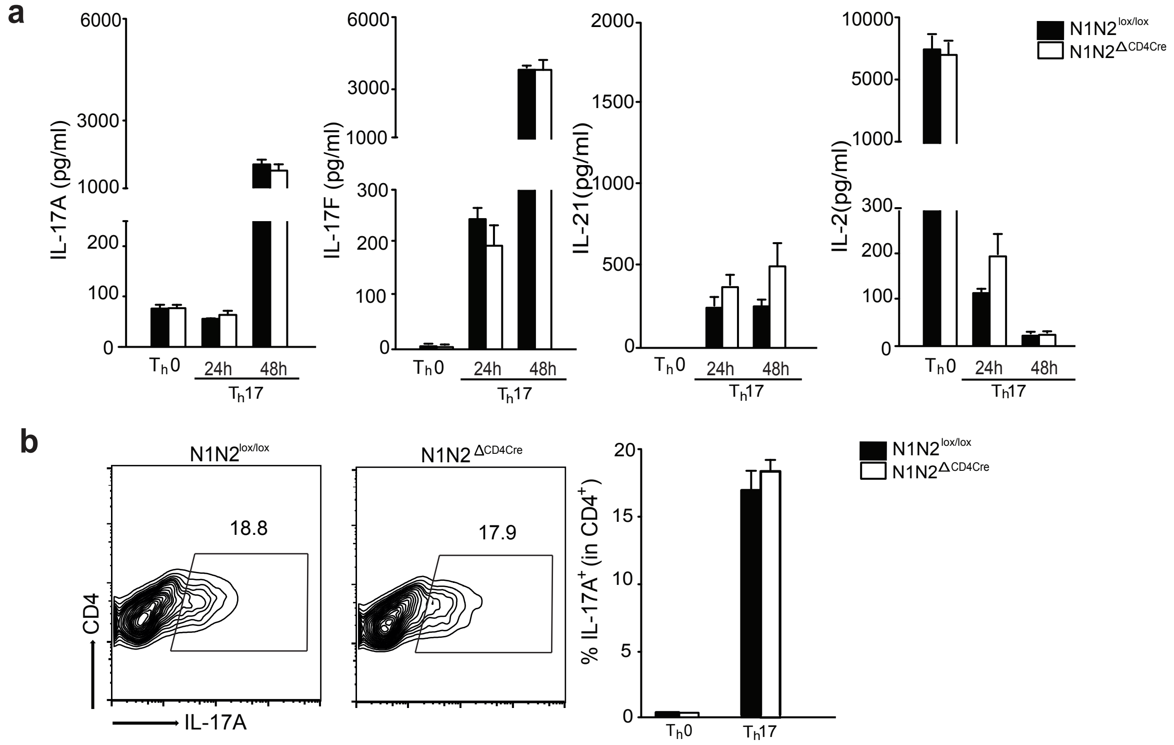
a
IL-17A (pg/ml)
IL-17F (pg/ml)
IL-21(pg/ml)
IL-2(pg/ml)
Th0
24h
48h
Th17
N1N2lox/lox
N1N2ΔCD4Cre
b
N1N2lox/lox
N1N2ΔCD4Cre
18.8
17.9
CD4
IL-17A
% IL-17A+ (in CD4+)
Th0
Th17
N1N2lox/lox
N1N2ΔCD4Cre
